# Downward movement of nitrate stimulates losses of soil organic carbon in deeper soil layers

**DOI:** 10.1073/pnas.2510350122

**Published:** 2025-06-13

**Authors:** Xiaotang Ju, Chong Zhang, Xue Tian

**Affiliations:** ^a^School of Tropical Agriculture and Forestry, Hainan University, Haikou 570228, China

**Fig. 1.**
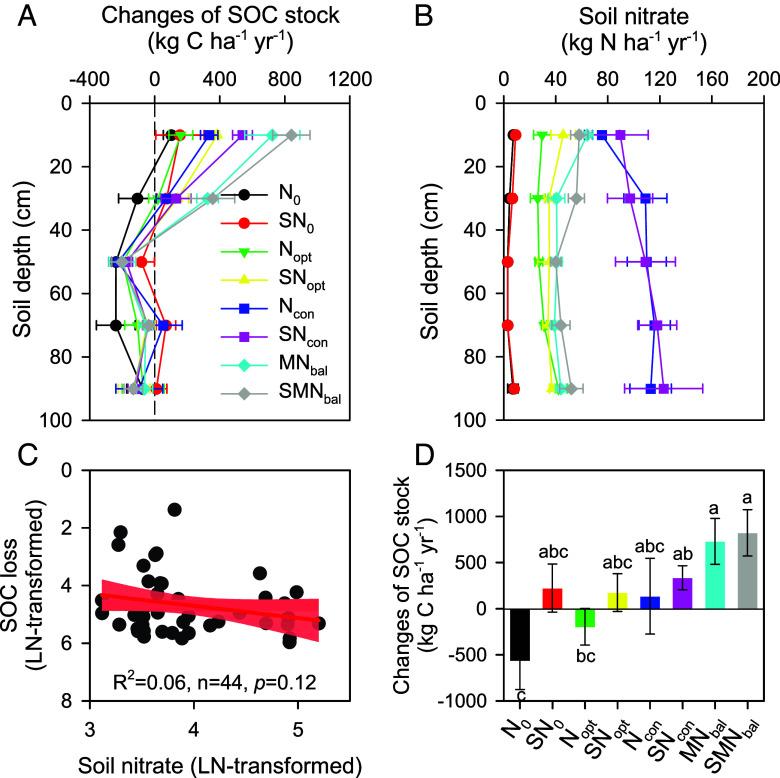
Changes of SOC stock and mean soil nitrate accumulation from 2006 to 2021 in a long-term field experiment. Changes of SOC stock (mean ± SE, n = 3) in each soil layer of the 0 to 100 cm soil profile in 2021 compared to 2006 (*A*). Mean soil nitrate accumulation (mean ± SE, n = 3) in each soil layer of the 0 to 100 cm soil profile from 2006 to 2021 (*B*). The correlation of the SOC loss to nitrate accumulation from fertilization treatments (expect for N_0_ and SN_0_ treatments) in the 40 to 100 cm soil layers (*C*); the red area around the regression line indicates the 95% CI. Changes of SOC stock in the 0 to 100 cm soil layers in 2021 compared to 2006 (*D*); the vertical lines are the SE (n = 3), different lowercase letters are significant differences between the treatments at *P* < 0.05. N_0_, N_opt_, N_con_, and N_bal_ are no N fertilizer, optimum N, conventional N, and balanced N rate, respectively. S and M denote straw and cattle manure, respectively. Soil nitrate was measured after the harvest of summer maize in October of each year. More detailed description of treatments can be found in Wei et al.’s work (2).

Zhou et al. (1) recently reported the dynamics of soil organic carbon (SOC) in China’s upland croplands from 1980 to 2023, with significant SOC accumulation in the upper 0 to 60 cm soil layers but with co-occurring SOC depletion in the 60 to 100 cm subsoil layers, based on large-scale resampling campaign (site by site). They attributed the subsoil SOC losses primarily to accelerated decomposition driven by climate warming over the past four decades using model-based analysis. Results from our 15-y long-term field experiment (2006 to 2021) corroborate this pattern of vertical SOC divergence (2), i.e., the net accumulation of SOC stock in the upper 0 to 40 cm soil layers but net losses in the deeper 40 to 100 cm soil layers (Fig. 1*A*). We further propose that another important cause of these losses is the positive priming effect induced by the downward movement of nitrate, which stimulates SOC decomposition in deeper soil layers. Our long-term field experiment provides direct evidence supporting this mechanism.

The long-term field experiment was established in 2006 with different carbon (C) and nitrogen (N) managements including eight treatments: zero-N fertilization without straw (N_0_) and with straw return (SN_0_), optimum synthetic N fertilization without straw (N_opt_) and with straw return (SN_opt_); conventional synthetic N fertilization without straw (N_con_) and with straw return (SN_con_), balanced organic with synthetic N fertilization without straw (MN_bal_) and with straw return (SMN_bal_) (2). We found declined SOC in 40 to 100 cm soil layers across all N fertilized treatments (Fig. 1*A*), and these reductions correlated with progressive nitrate accumulation observed over the 15-y experimental period (Fig. 1 *B* and *C*). Nitrite-induced positive priming accelerated SOC decomposition beyond formation rates in deeper soil layers that received fewer crop residues than upper layers (3–5). The smaller SOC loss in the 40 to 100 cm layers under SN_0_ compared to N_0_ further supports this, as N limitation led to more residue retention and less SOC decomposition (Fig. 1*A*) (4, 5). Nitrate migration has been shown to intensify SOC losses even at depths of 2 to 12 m (6). In N-fertilized croplands, nitrate serves as an alternative electron acceptor, alleviating N constraints on SOC decomposers and enzymes and triggering positive priming under anoxic conditions (3, 6, 7).

The results of our long-term field experiment also clearly demonstrate the measures for building up the SOC stock in the entire 0 to 100 cm soil profile. Long-term balanced organic with synthetic N fertilization can largely increase SOC stocks, and the straw return combined with optimal N fertilization is also essential for maintaining and increasing SOC stocks throughout the 0 to 100 cm soil profile in the long run (Fig. 1*D*).
